# Letter from the Editor in Chief

**DOI:** 10.19102/icrm.2025.16072

**Published:** 2025-07-15

**Authors:** Devi Nair


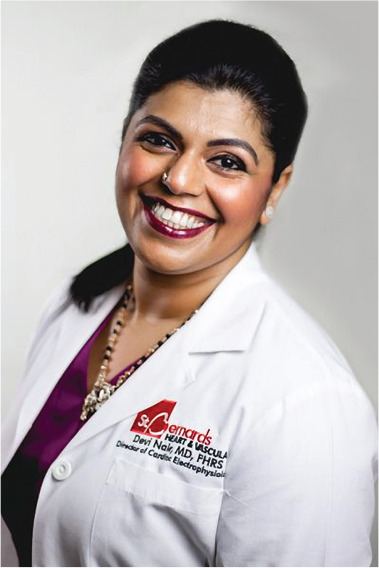


Dear Colleagues,

As we arrive in the heart of the summer season, our field continues to surge forward with innovation, insight, and an ever-growing spirit of collaboration. This July issue of *The Journal of Innovations in Cardiac Rhythm Management* reflects the dynamic landscape of cardiac electrophysiology, where cutting-edge science and clinical integration go hand in hand and feature a spectrum of clinical science—from nuanced arrhythmia mapping to anticoagulation strategies in complex structural settings that reflect both the technical progress and the collaborative spirit that increasingly define our field.

This month’s manuscripts represent both core rhythm challenges and the expanding reach of our specialty:

- Khan et al.^1^ present a comprehensive umbrella review evaluating direct oral anticoagulants (DOACs) versus vitamin K antagonists in patients with atrial fibrillation undergoing bioprosthetic valve replacement. Their findings support the increasing use of DOACs in this population, offering reassurances of safety and thromboembolic protection.
- Harmouch et al.^2^ contribute three compelling case reports demonstrating the value of electrogram analysis during ventricular tachycardia ablation, illustrating how careful interpretation of local signals can drive real-time termination and targeted substrate modification in complex ventricular arrhythmias.
- Yorgun et al.^3^ introduce a novel method of functional substrate mapping in the right atrium, demonstrating that deceleration zones identified during sinus rhythm correspond with critical isthmus sites for re-entrant atrial tachycardia. Their findings may help shape ablation strategies for complex right atrial arrhythmias in patients with scarring.
- Kean et al.^4^ provide a sobering analysis of the association between inappropriate implantable cardioverter-defibrillator (ICD) shocks and increased mortality in patients with ventricular arrhythmias. Their single-center registry emphasizes the critical need for continued refinement in ICD programming and patient monitoring.

## A Season of Transition in Training

July also marks a pivotal month for our community on a personal level. Across the country and around the world, we welcome a new class of electrophysiology fellows, ready to begin their journey into the subspecialty. We also celebrate the achievements of graduating senior fellows, many of whom are stepping into independent practice as the next generation of academic leaders, procedural experts, and advocates for patients.

To support this crucial transition, August will feature a number of fellow-focused educational events, including the annual EP Fellows Conferences in Austin and Kansas City. These immersive programs provide not just procedural training and academic learning but also invaluable opportunities for mentorship, collaboration, and professional development.

## Your Voice in the Rhythm Community

As the journal continues to evolve alongside the field, we invite you to become a part of its voice. Whether you’re a practicing electrophysiologist, a trainee, an imager, a structural colleague, or a heart rhythm nurse or tech, we welcome your submissions of original research, insightful cases, early experiences, or commentary.

This journal exists as a platform to reflect the full breadth of our community, and it thrives on the exchange of ideas from every level and background. We especially encourage our newest fellows and early-career colleagues to share their unique perspectives as they train, transition, and grow within the field.

In a time of rapid innovation, *The Journal of Innovations in Cardiac Rhythm Management* remains committed to being not just a record of what’s happening but also a place where knowledge is shared, mentorship begins, and the rhythm community connects.

We hope this issue offers insight and inspiration as you continue your work in the lab, the clinic, and the community. Whether you are mentoring new fellows or reflecting on your own evolution as a practitioner, we thank you for your contributions to this dynamic and ever-growing field.

Warm regards,


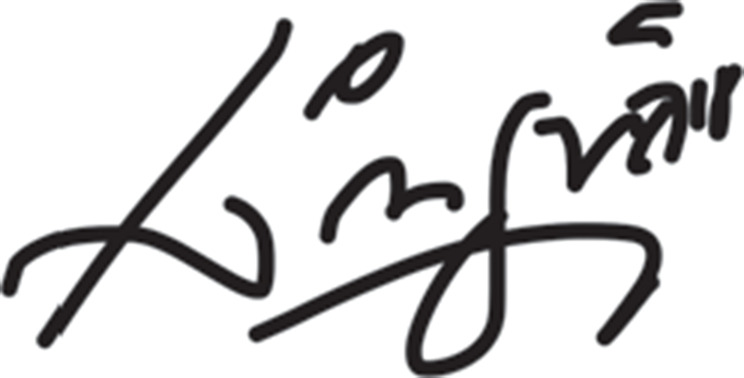


Dr. Devi Nair, md, facc, fhrs

Editor-in-Chief

*The Journal of Innovations in Cardiac Rhythm Management*

Director of the Cardiac Electrophysiology & Research,

St. Bernard’s Heart & Vascular Center, Jonesboro, AR, USA

White River Medical Center, Batesville, AR, USA

President/CEO, Arrhythmia Research Group

Clinical Adjunct Professor, University of Arkansas for Medical Sciences

Governor, Arkansas Chapter of American College of Cardiology

drdgnair@gmail.com
